# Biosensing approaches in body fluids using extended-gate-type organic field-effect transistor enzymatic sensors

**DOI:** 10.1007/s44211-025-00750-8

**Published:** 2025-04-05

**Authors:** Yui Sasaki, Tsuyoshi Minami

**Affiliations:** 1 Research Center for Advanced Science and Technology, The University of Tokyo, 4-6-1 Komaba, Meguro-Ku, Tokyo, Japan; 2 Institute of Industrial Science, The University of Tokyo, 4-6-1 Komaba, Meguro-Ku, Tokyo, Japan; 3 JST, PRESTO, 4-1-8 Honcho, Kawaguchi, Saitama Japan

**Keywords:** Organic field-effect transistor, Extended gate, Biosensor, Enzyme, Body fluid

## Abstract

Biomarkers in body fluids provide essential chemical information for examining health conditions; however, unlike conventional instrumental approaches, easy-to-use analytical methods have not yet been fully established. This review introduces extended-gate-type organic field-effect transistors (OFETs) as biosensor platforms for real-sample analysis. OFETs are electronic devices that show switching profiles when gate voltages are applied. Therefore, the gate electrode of OFET functions as a sensing unit combined with appropriate molecular recognition materials. Owing to their signal amplification properties, OFETs enable sensitive biosensing. The extended-gate surfaces are easily functionalized with enzymatic layers using chemical modification, and these surfaces provide a high discrimination ability for specific biomarkers from their analogs. This review presents the designs of the extended-gate structures (i.e., integrated and separated styles) and their enzymatic layers and includes their actual sensing performance.

**Graphical abstract:**

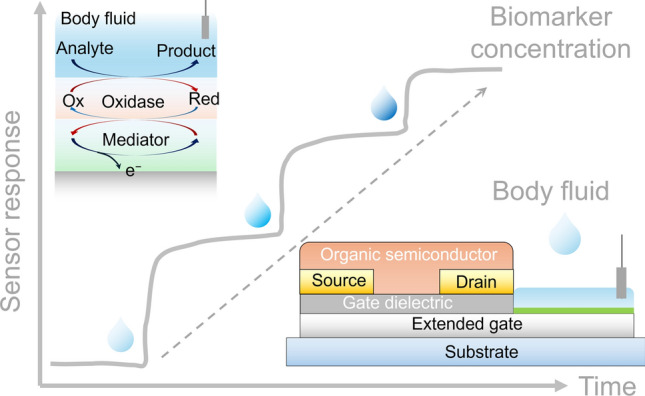

## Introduction

Chemical species contained in body fluids are essential biomarkers for examining health conditions, and abnormal levels indicate significant diseases [1]. Stational analytical equipment has been widely used for the assessment of body fluids because of their reliability and accuracy. However, conventional instrumental approaches have requirements of complicated sample treatment, trained personnel, large apparatuses, and long-term measurements. Therefore, portable sensor devices are potential candidates for real-sample analysis in practical situations.

Sensors consist of receptors and transducers, and the combination of both units defines the sensor abilities such as sensitivity, selectivity, and response time [2]. Enzymes are representative biogenic recognition materials that allow selective analyte capture based on the lock-and-key detection principle [3]. Enzymes have already been applied as blood glucose sensors for diabetes [4]. Although these conventional biosensors have been used in real-world scenarios, several drawbacks exist. For example, an enzymatic glucose sensor requires the collection of blood samples and causes pain to the patients. Therefore, ideal biosensors require not only selective and sensitive detectability but also the ability to painlessly collect body fluids. Among body fluids, with the exception of blood, urine, sweat, saliva, and exudates are collected painlessly [1]. In this context, glucose concentrations are at millimolar levels in blood samples [5], whereas sensitive detection at micromolar levels is required in sweat samples [6–11]. Specifically, biomarker concentrations depend on the type of body fluid, which indicates that the selection of appropriate transducers is needed for each target sample.

Field-effect transistors (FETs) are electronic devices that consist of three electrodes (i.e., gates, sources, and drains) and dielectric and semiconductor layers [12]. FETs display current switching profiles corresponding to applied gate voltages; here, the gate electrode performs as a sensing site by integrating appropriate molecular recognition materials (vide infra) [13–15]. In the design of chemical/biosensors, appropriate sensitivity needs to be considered depending on analyte concentrations. To date, amperometric enzymatic sensors [16] or colorimetric enzyme-linked immunosorbent assays [17] have been applied for the sensitive detection of biomarkers. The superior features of FETs over conventional detection methods in chemical/biosensing include signal amplification abilities [18] and the applicability of label-free and real-time measurements. Clearly, the favorable device properties of FETs have been vigorously expanded for sensor applications [19, 20]. However, among semiconductive materials, the instability of organic semiconductors in water environments has posed the development of organic FET (OFET)-based chemical/biosensors that require their use in aqueous media [21]. To overcome the drawbacks of OFETs, the focus of this study is on an extended-gate structure for chemical sensing [22–26]. The OFET device allows monitoring of time- and concentration-dependent enzymatic reactions on the gate electrode when changes in transistor characteristics occur upon analyte capture. This review summarizes the designs of OFET devices and gate electrode surfaces for sensing in aqueous solutions and their biosensor performance in human urine and sweat samples.

## Principle of extended-gate-type organic FETs for biosensing

The extended-gate structure is a promising device configuration for obtaining stable sensor signals corresponding to biomarker detection in body fluids. One of the pioneering works regarding an extended-gate FET was reported by Van der Spiegel and coauthors in 1983 [22]. In this report, a gate electrode of a metal‒oxide‒semiconductor FET (MOSFET) served as a sensing unit that was functionalized with films made of IrO_X_, LaF_3_, or AgCl. The sensing unit was integrated with the MOSFET on a silicon substrate as a single chip, and the chip could collect the potentiometric measurements of chloride (Cl^−^) and fluoride (F^−^) ions and protons (H^+^). After three decades, another extended-gate structure was reported as an OFET-based biosensor. Biosensors for body fluids need to possess not only robustness in assessment environments but also the disposability of the sensing units owing to hygiene aspects in practical use. To this end, Minami et al. fabricated an extended-gate electrode on a flexible polyethylene naphthalate (PEN) film, and arranged to separate it from the OFET unit [23]. Overall, these works clarified the feasibility of extended-gate FET structures for biosensing.

**Fig. 1:**
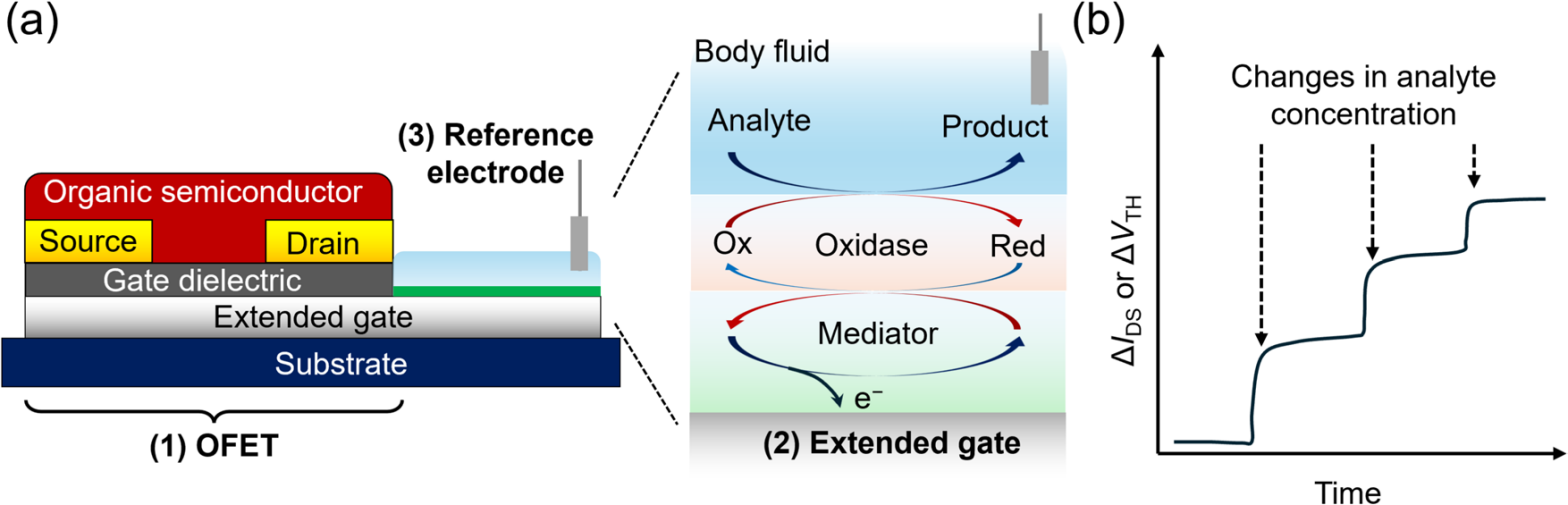
**a** Schematic illustration of an extended-gate type OFET for biosensing. The extended-gate surface is functionalized with an enzymatic layer. **b** Time-dependent changes in the transistor characteristics of the drain current (*ΔI*_DS_) or threshold voltage (*ΔV*_TH_) with varying analyte concentration

The extended-gate FET sensors generally consist of (1) FET devices, (2) sensing gate electrodes, and (3) reference electrodes, and the arrangements of each element are changed according to the sensing purpose (Fig. 1a). The operation principle of OFETs is in accordance with that of MOSFETs. The details of FET operation have been summarized in various review articles [12, 26]; therefore, we describe the principle of extended-gate-type OFETs for biosensing. As shown in Fig. 1a, the extended-gate electrode is functionalized with an enzymatic layer to detect analyte information through the changes in transistor characteristics. The addition of an analyte in an aqueous solution induces an enzymatic reaction accompanied by product generation, which causes electron relay on the extended-gate electrode through a mediator layer. The change in the surface potential of the sensing gate electrode is subsequently caused by the electron relay, followed by the influence of the conductance of the OFETs. The sensor responses of OFETs upon biosensing are expressed as varying drain currents (*I*_DS_s) and threshold voltages (*V*_TH_s). The reference electrode is used to apply a certain gate voltage (*V*_GS_); thus, the extended-gate OFET sensors depend on potentiometric detection. The relationships among the above factors can be expressed by Eq. (1):

1 $$I_{{{\text{DS}}}} = { }\left( \frac{W}{2L} \right)\mu C\left( {V_{{{\text{GS}}}} - V_{{{\text{TH}}}} } \right)^{2}$$

where the terms *W* and *L* are the channel width and length of the OFET device, respectively, and the terms *μ* and *C* are the field-effect mobility and the capacitance of the gate dielectric, respectively. Moreover, biosensing information detected at the interface between the enzymatic gate electrode and the aqueous solution containing analytes can be expressed using *V*_TH_ of the OFET as follows:

2 $$\Delta V_{{{\text{TH}}}} = \frac{{{\Delta }Q}}{C}$$

where the *V*_TH_ change (Δ*V*_TH_) is induced by varying the charge density *Q* (Δ*Q*) according to the electron relay [27]. Consequently, time- and analyte concentration-dependent enzymatic reactions can be detected as shifts in the transistor characteristics, such as Δ*I*_DS_ and Δ*V*_TH_ (Fig. 1b).

### Integrated-type extended-gate organic FET biosensors

**Fig. 2:**
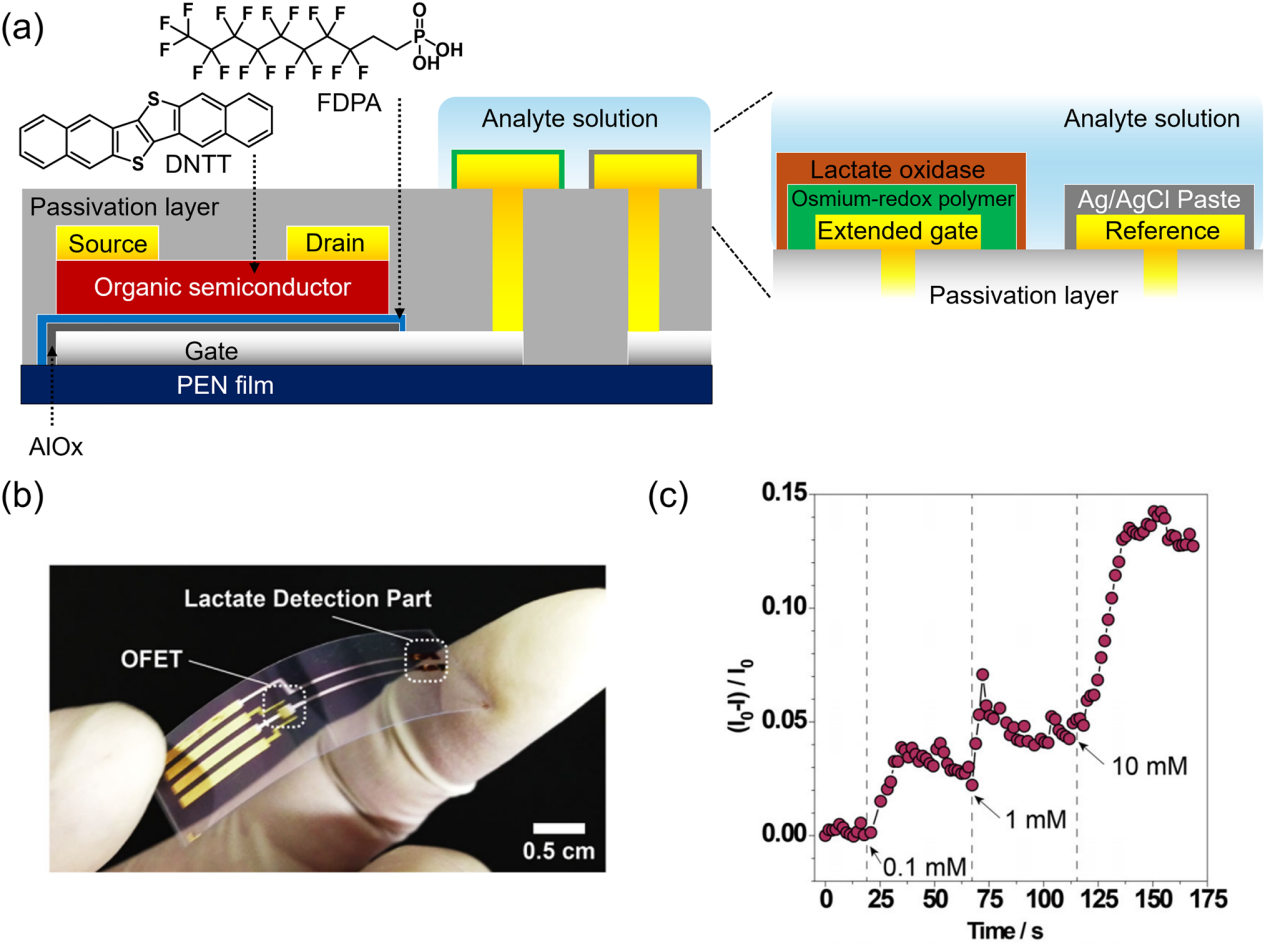
**a** Schematic illustration of the integrated type of OFET-based biosensor for lactate sensing and the chemical structures of device components. **b** Photograph of the fabricated OFET-based biosensor. **c** Time-course changes in *I*_DS_ upon the addition of lactate. The terms *I*_DS0_ and *I*_DS_ indicate the drain currents of the biosensor before and after the addition of lactate. Reproduced with permission from reference 32. 2019 Springer Nature

The focus of this section is on the biosensor performance of an integrated structure for extended-gate-type OFETs. The target lactate is a representative biomarker detected in human sweat, and monitoring sweat lactate is essential, especially for athletes, because the lactate levels are correlated with physical performance [28–31]. Therefore, a low-voltage operational biosensor on a flexible substrate is desirable for real-time monitoring in sports science fields. In this study, an OFET unit and a sensing electrode were fabricated on one chip made of a PEN film (Fig. 2a, b) [32]. The dielectric layers of the OFET were constructed of aluminum oxide and 1*H*,1*H*,2*H*,2*H*-perfluoro-*n*-decylphosphonic acid (FDPA). The thin dielectric layer contributed to low-voltage operation below |3| V. Dinaphtho[2,3-*b*:2’,3’-*f*]thieno[3,2-*b*]thiophene (DNTT) was used as the organic semiconductive layer. The surface of the device chip was entirely covered with a perylene layer for passivation. The extended sensing unit and a reference electrode were formed in parallel using Au on the perylene layer. The sensing unit was connected to the gate electrode of the OFET via holes. The reference Au electrode was covered with an Ag/AgCl paste. The sensing electrode surface was subsequently functionalized with a horseradish peroxidase osmium-redox polymer (as a bottom layer) and lactate oxidase (as a top layer) to detect the target lactate by enzymatic catalysis. As shown in Fig. 2c, the fabricated biosensor showed time-dependent *I*_DS_ changes upon the addition of different lactate concentrations. In the enzymatic reaction on the extended-gate electrode, pyruvate and hydrogen peroxide are produced from lactate and oxygen in aqueous media by lactate oxidase. Lactate oxidase further reacts with the produced hydrogen peroxide, which generates water molecules. The enzymatic reaction by lactate oxidase causes valence changes in the osmium ions in the redox polymer layer, resulting in varying channel conductance of the OFET [33, 34]. Overall, the results reveal that the OFET can successfully read out a time- and analyte concentration-dependent electron relay that occurred on the extended-gate electrode.

### Separated-type extended-gate organic FET biosensors

In biosensor devices, especially sensing electrodes, hygiene aspects need to be considered for real-sample analysis of human body fluids. For this purpose, the separated structure of the extended-gate sensing electrode from the OFET is crucial for its disposability. In this section, extended-gate-type OFET-based biosensors are described based on a separated structure for accurate real-sample analysis [11, 35].

**Fig. 3:**
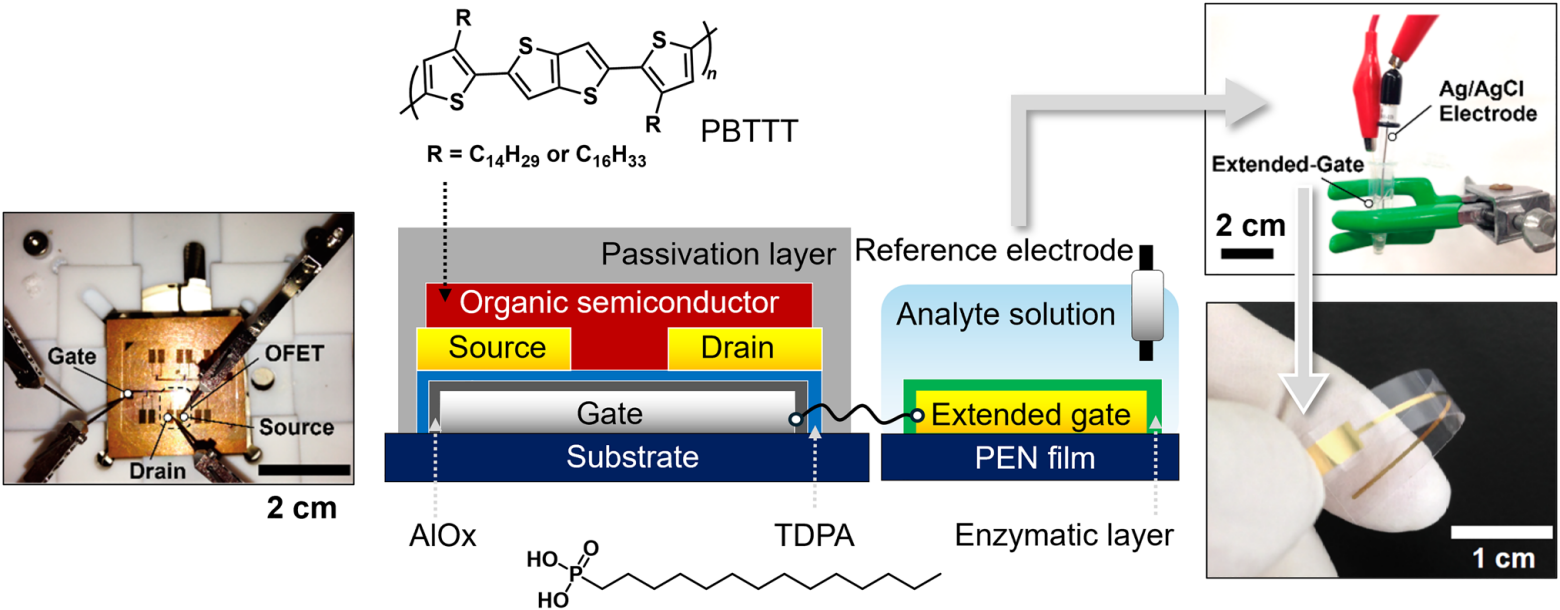
Schematic illustration and photographs of a separated-type extended-gate OFET-based biosensor and the chemical structures of device components. Reproduced with permission from reference 38. 2016 Multidisciplinary Digital Publishing Institute

The OFET device was designed to achieve low-voltage operation for biosensing in body fluids. The combination of an aluminum oxide layer and a tetradecylphosphonic acid (TDPA) layer served as the gate dielectric layer [36]. The organic semiconductive layer was formed by drop-casting a solution-processable π-conjugated polymer material (i.e., poly{2,5-bis(3-alkylthiophen-2-yl)thieno[3,2-*b*]thiophene} (PBTTT)) [37]. A fluorinated polymer material was fully coated on the device surface for passivation. The extended-gate Au electrode was fabricated on a PEN film, which was connected to the gate electrode of the OFET unit through a conductive cable. The gate voltages were applied through the reference electrode (Ag/AgCl) in OFET operation. The extended-gate electrode and the reference electrode were immersed in analyte solutions (such as body fluids) to perform potentiometric measurements at the interfaces between the sensing electrode and the analyte solutions (Fig. 3). [38]

In the design of enzymatic electrodes, the construction of uniform enzymatic layers is necessary to obtain accurate sensor signals derived from the electron relays on the extended-gate electrodes. Herein, two sensing examples for dopamine and glucose are introduced as approaches using self-assembled linked mediator layers.

**Fig. 4:**
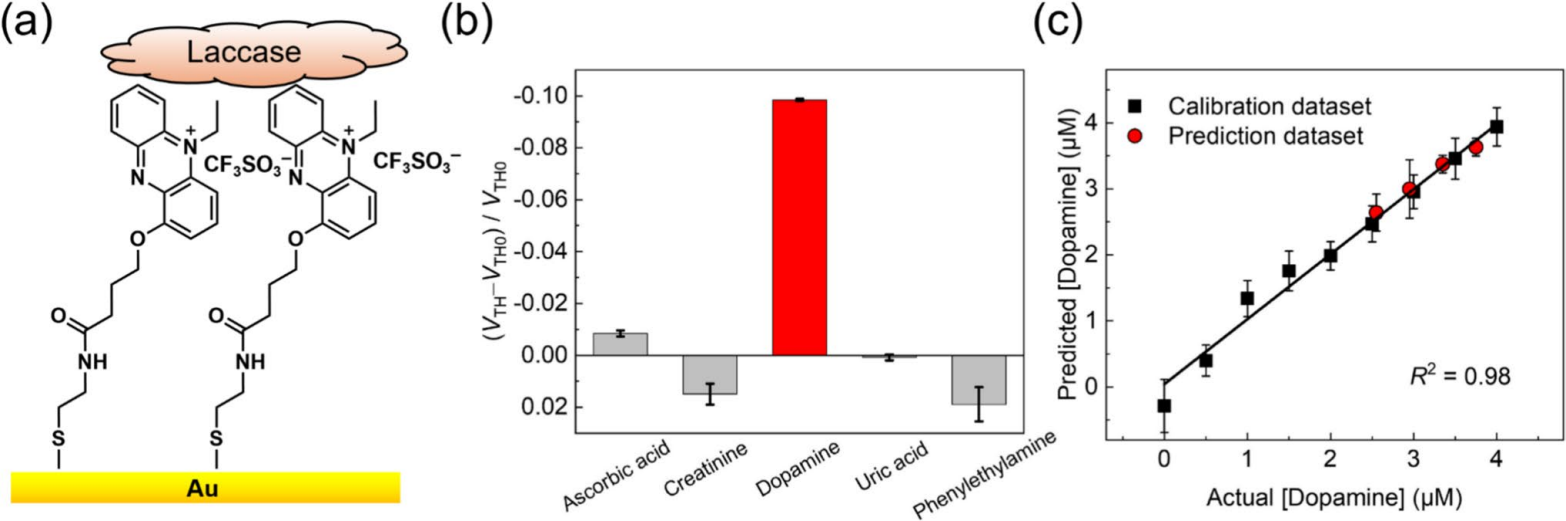
**a** Schematic illustration of the extended-gate surface functionalized with a SAM linked to an *N*-ethylphenazonium moiety and laccase. **b** Selectivity test results for the five analytes (50 μM). The terms *V*_TH0_ and *V*_TH_ indicate threshold voltages before and after adding analytes. **c** Spike recovery test for dopamine in a human urine sample without any pretreatment (ethics authorization code: 22–235). Reproduced from reference 35. Copyright 2023 Elsevier

Catecholamines such as dopamine, adrenaline, and noradrenaline play crucial roles as neurotransmitters in the sympathoadrenal system [39, 40]. Changes in the concentrations of these catecholamines influence the activity of neurotransmitters, causing mental disorders [41]. In addition, catecholamines are generated by tumors in abnormal states, which leads to pheochromocytomas and paragangliomas. In this context, noradrenaline- and adrenaline-secreting pheochromocytomas show clinical symptoms, including panic attacks, elevated blood pressure, and migraine headaches, whereas no symptoms are observed in dopamine-secreting tumors [41]. Therefore, dopamine sensing in urine is beneficial for the early detection of asymptomatic diseases. In urinalysis, electrochemically active interferents such as uric acid and ascorbic acid cause noise signals because of their oxidation from the application of a high potential to a sensing electrode [43, 44]. Moreover, high discrimination ability of dopamine from adrenaline and noradrenaline is needed for accurate urinalysis, even though the actual concentrations of the analog are much lower than those of dopamine [42]. Therefore, the enzymatic catalysis of laccases is a promising approach for selective dopamine detection. Considering this, a combination of an *N*-ethylphenazonium moiety-linked SAM and a laccase (from *Trametes versicolor*) was applied to detect urinary dopamine [43–45]. The selected *N*-ethylphenazonium moiety for the mediator unit provides a low redox potential, which avoids unexpected interferent effects originating from electrochemically active species. In this assay, the thiolated mediator unit was immobilized through a thiol‒Au bond to uniformly perform an electron relay of enzymatic catalysis on the extended-gate electrode (Fig. 4a). Compared with other electrochemical interferents, such as ascorbic acid, creatinine, uric acid, and phenylethylamine, the enzymatic OFET sensor showed the highest response to dopamine (Fig. 4b). In this regard, the estimated limit of detection for dopamine (0.19 μM) indicated the potential of the OFET-based biosensor for urinalysis [46]. Notably, the obtained sensitivity of the OFET-based biosensor was greater than that of conventional electrochemical sensors (i.e., differential pulse voltammetry) [47, 48]. A spike-and-recovery test was subsequently performed to evaluate the applicability of the OFET-based biosensor for practical analysis without sample pretreatment. In this assay, a calibration line for dopamine was established by collecting the changes in the transistor characteristics at different concentrations of dopamine. As shown in (Fig. 4c), four datasets (red circles) corresponding to dopamine in human urine were distributed on the calibration line. The recovery rates of all four datasets were estimated to be 97–104% at 2.55–3.75 μM; these values were comparable to those of a clinical urinalysis instrument.

**Fig. 5:**
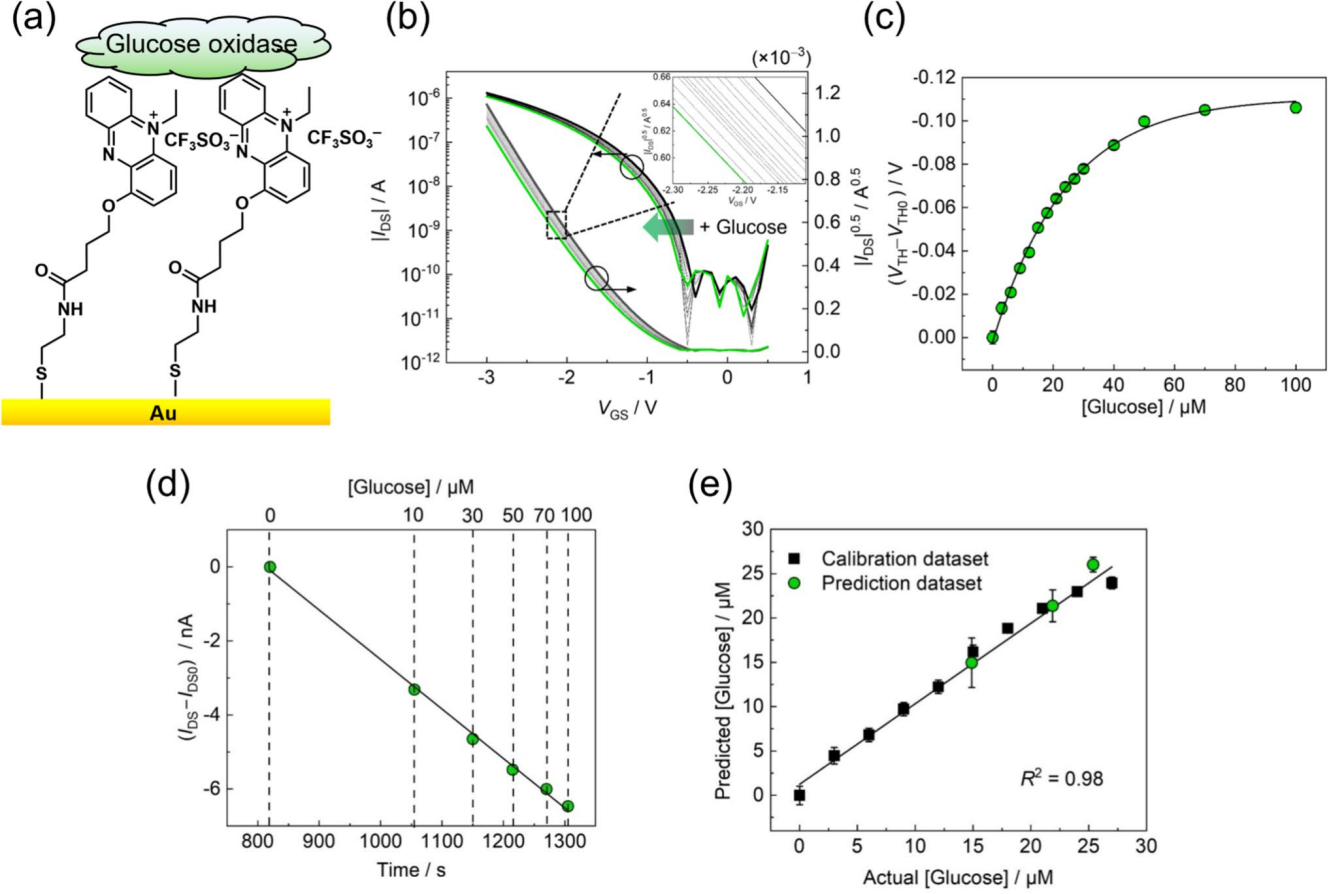
**a** Schematic illustration of the extended-gate surface functionalized with a SAM linked to an *N*-ethylphenazonium moiety and glucose oxidase. **b** Shifts in the transistor characteristics of the enzymatic sensor upon the addition of glucose (0 − 100 μM). **c** Titration isotherm for glucose. The terms *V*_TH0_ and *V*_TH_ indicate threshold voltages before and after the addition of glucose. **d** Time- and concentration-dependent *I*_DS_ changes based on glucose detection. The terms *I*_DS0_ and *I*_DS_ represent drain currents before and after the addition of glucose. **e** Spike and recovery test for glucose in a human sweat sample without any pretreatment (ethics authorization code: 22–244). Reproduced from reference 11. Copyright 2024 Wiley–VCH GmbH

The next target biomarker is glucose in human sweat. In contrast to blood glucose, sweat glucose exists at micromolar levels [6–11]. To achieve sensitive and selective glucose detection in human sweat, the combination of the SAM-linked *N*-ethylphenazonium moiety [35], a coenzyme (flavin adenine dinucleotide (FAD)), and glucose oxidase (from *Aspergillus niger*) was employed for the construction of the enzymatic electrode (Fig. 5a). The transistor characteristics of the enzymatic sensor were quantitatively shifted by the addition of glucose (Fig. 5b). The limit of detection value was estimated to be 2.9 μM based on the 3σ method (Fig. 5c). The LoD value of the OFET-based biosensor was clearly lower than that of conventional amperometric [49] and colorimetric enzymatic sensors [7]. Notably, the sensitivity of the OFET-based glucose sensor met the requirements for practical glucose sensing in sweat [6]. In addition, the extended-gate OFET showed continuous *I*_DS_ changes depending on the glucose concentration. An 80% response to glucose was reached at 15 s after glucose addition (100 μM). Figure 5d shows a linear dependency between the response time of the enzymatic reaction and the glucose concentration. Finally, glucose detection in human sweat without pretreatment was carried out using the OFET-based sensor. The accuracy of the enzymatic sensor was evaluated using a reliable analytical instrument (HPLC). The obtained spike-and-recovery rates for glucose were 95–105% (Fig. 5e); these results revealed the applicability of the extended-gate-type OFET for sweat glucose sensing with high accuracy.

## Conclusion

Body fluids contain essential biomarkers, which provide vital chemical information for examining health conditions. Conventionally, invisible biomarkers in body fluids have been assessed using reliable instrumental methods; however, these techniques are not suitable for rapid onsite analysis. To establish analytical methods for real samples, organic field-effect transistor (OFET)-based enzymatic sensors were employed as platforms for the selective and sensitive analyte detection in body fluids. The device characteristics of OFETs are modulated using gate voltages; thus, the quantitative shifts in drain currents or threshold voltages provide biosensing information detected on a gate electrode. The focus of this review is on real-sample analysis using extended-gate-type OFET-based enzymatic sensors. The extended-gate structures are classified into integrated and separated fashions, and their gate elements are functionalized with enzymatic layers depending on target biomarkers. In this review, two device configurations and their sensor performances are summarized. Although OFET-based enzymatic sensors have revealed favorable discrimination ability for specific biomarkers from interferents owing to the lock-and-key detection principle of enzymes, the instability of the biogenic recognition materials to chemical and physical stimuli is a concern in practical sensing situations. To overcome this difficulty, robust synthetic receptors designed based on molecular recognition chemistry are potential materials [16, 50]. Overall, we describe the potential of the extended-gate structures for real-sample analysis. Moreover, other device configurations using gate surfaces (e.g., top-gate structures) can also be applied for sensing in aqueous media and could facilitate sensor development owing to the simplified structures from the viewpoint of device fabrication 51. As shown above, some improvement is needed for realistic biomarker sensing; however, our study has shown that the combination of OFETs and appropriate molecular recognition materials enables accurate real-sample analysis comparable to that of conventional instrumental methods.

## Data Availability

This review article does not contain separate datasets.
